# In-Season Predictions Using Chlorophyll *a* Fluorescence for Selecting Agronomic Traits in Maize

**DOI:** 10.3390/plants14081216

**Published:** 2025-04-15

**Authors:** Andrija Brkić, Sonja Vila, Domagoj Šimić, Antun Jambrović, Zvonimir Zdunić, Miroslav Salaić, Josip Brkić, Mirna Volenik, Vlatko Galić

**Affiliations:** 1 Agricultural Institute Osijek, 31000 Osijek, Croatia; domagoj.simic@poljinos.hr (D.Š.); antun.jambrovic@poljinos.hr (A.J.); zvonimir.zdunic@poljinos.hr (Z.Z.); miroslav.salaic@poljinos.hr (M.S.); josip.brkic@poljinos.hr (J.B.); mirna.volenik@poljinos.hr (M.V.); 2 Department for Plant Production and Biotechnology, Faculty of Agrobiotechnical Sciences Osijek, Josip Juraj Strossmayer University of Osijek, 31000 Osijek, Croatia; sonja.vila@fazos.hr

**Keywords:** indirect selection, maize, chlorophyll fluorescence

## Abstract

Traditional maize (*Zea mays* L.) breeding approaches use directly measured phenotypic performance to make decisions for the next generation of crosses. Indirect assessment of cultivar performance can be utilized using various methods such as genomic predictions and remote sensing. However, some secondary traits might expand the breeder’s ability to make informed decisions within a single season, facilitating an increase in breeding speed. We hypothesized that assessment of photosynthetic performance with chlorophyll *a* fluorescence (ChlF) might be efficient for in-season predictions of yield and grain moisture. The experiment was set with 16 maize hybrids over three consecutive years (2017–2019). ChlF was measured on dark-adapted leaves in the morning during anthesis. Partial least squares models were fitted and the efficiency of indirect selection was assessed. The results showed variability in the traits used in this study. Genetic correlations among all traits were mainly very weak and negative. Heritability estimates for all traits were moderately high to high. The model with 10 latent variables showed a higher predictive ability for grain yield (GY) than other models. The efficiency of the indirect selection for GY using biophysical parameters was lower than direct selection efficiency, while the indirect selection efficiency for grain moisture using biophysical parameters was relatively high. The results of this study highlight the significance and applicability of the ChlF transients in maize breeding programs.

## 1. Introduction

In modern maize breeding, the most important traits are grain yield and grain moisture (GM) at harvest time [1]. Since these two traits are quantitative traits with low heritability, an alternative option for the breeders is to find secondary traits (STs), which provide additional information about plant status and yield in various environments and thus enable indirect selection for traits of interest [2]. In order to utilize STs in the selection process, they need to be genetically stable, correlated to yield, simple and cheap in terms of measurements, and with high heritability scores [3,4]. Moreover, STs need to be recorded in flowering time or before and showcase the yield potential long before the harvest. Some STs include the anthesis–silking interval (ASI), leaf and stalk elongation, leaf temperature, leaf rolling, leaf position, leaf chlorophyll concentration, stay green trait, tassel size, leaf osmotic concentration, and lodging [5].

The indirect selection proved to be useful in breeding for stress-related traits such as drought, nitrogen deficiency, and disease tolerance [6,7,8,9]. Therefore, maize breeders exploit multiple morphological, physiological, and biochemical traits in selection programs in order to develop a more complete perspective of the whole process. Additional information coming from secondary traits allows breeders to run their programs with more efficiency and to develop inbred lines and hybrids. The efficiency of indirect selection has been studied extensively [10,11,12,13,14], highlighting traits such as drought tolerance, soil water surplus, photosynthetic efficiency, soil nitrogen deficiency, chilling tolerance, and many others. There is a strong linear association between the flowering date (FD) and GY, FD and GM, and GM and GY [15]; therefore, the most common secondary traits in maize breeding are traits associated with maize flowering time, i.e., pollination date, silking date, and ASI [16]. However, continuous selection for specific secondary traits varies not only their mean value but also modifies their genetic correlation with yield [17], and can potentially affect the genetic gain. Consequently, associations between secondary traits and GY require re-evaluation after a certain period [5].

Chlorophyll fluorescence analysis is one of the most common techniques for evaluating the effect of stress on photosynthesis. The F_v_/F_m_ parameter [18] was widely used as an indicator of the photosynthetic efficiency in plants. A decrease in this index indicates lower efficiency of the photosystem II (PSII). In the last two decades, ChlF applications have advanced extensively, and many other new parameters for measuring PSII efficiency have been designed [19]. After the testing of various fluorescence techniques, two main techniques prevailed, direct [20] and modulated fluorescence [21], of which the former comprises certain advantages such as collecting more information during measurement, more independence in fieldwork, a large number of tested samples, and generating data in all weather conditions [22]. Other techniques include pump and probe fluorometry [23], fast repetition rate (FRR) [24], and pump during probe fluorometry [25]. Tolerance of crops to stress conditions is one of the main goals in research and breeding, and ChlF measurements during drought or high-temperature scenarios are a valuable tool for biologists, ecophysiologists, and breeders [26].

Although ChlF is—due to the simplicity of its use and cost-effectiveness—one of the widely used ecophysiological techniques for studying photosynthesis in plants [27], data interpretation and theory behind ChlF still constitute a complex domain. Numerous reviews report the theoretical background of the ChlF analysis, with a methodology for generating information and clarifying physiological mechanisms associated with photosynthesis [28,29,30,31,32,33,34,35]. PSII is a component of the thylakoid membranes of chloroplasts that is most sensitive to damage, thereby the main result of the abiotic stress is to induce photoinhibition of PSII [36]. On the other hand, the protective mechanism of the photosystem I (PSI) can prevent photoinhibition, thus ensuring less damage [37]. The key parameter for detecting the photoinhibition of PSII caused by stress factors is F_v_/F_m_ [38], which is the maximum quantum yield of PSII and one of the most applied parameters in chlorophyll *a* fluorescence measurements [39].

Using a ChlF in maize breeding can provide valuable information for indirect selection programs. Various studies have explored the use of ChlF in maize breeding, such as an analysis of the genetic variability and detection of potential quantitative trait loci (QTL) for two ChlF parameters in a maize IBM population (intermated B73 × Mo17) [40] under different water availability conditions [41] and an exploration of the effect of drought stress and excess cadmium stress on photosynthetic activity in maize using chlorophyll *a* fluorescence [42]. Analysis of agronomic traits in maize such as plant density, drought stress, and cold and heat stress using ChlF methods is an additional source of information about yield and other yield-related traits significant for maize selection [43,44,45,46]. An evaluation of the yield in drought conditions through ChlF reported that using solar-induced fluorescence (SIF) is more reliable than other applied methods [47], which was confirmed by other studies [48]. A study evaluating genetic correlations between ChlF and GY in different temperature scenarios [49] reported that performance index on absorption basis (PI_ABS_) could be an effective parameter in maize breeding in moderate heat conditions, considering their genetic correlation with GY.

Various statistical and machine learning methodologies are used to predict hybrid performance in maize in different scenarios [50,51]. Predictions of important agronomic traits such as GY have become valuable tools for maize breeding programs in the last two decades.

The main hypothesis of this study is that variability in fluorescence traits and their genetic correlation to grain yield and moisture can facilitate in-season predictions of maize performance. GY and GM predictions through fluorescence traits in pollination time would enable the realization of pollination tasks in the actual season (crossing and selfing), thus developing the superior progenies that would shorten the hybrid development cycle and ensure more efficient resource management.

## 2. Results

### 2.1. Mean Values and ANOVA for GY and GM, F_v_/F_m_, and PSI_ABS_

**Figure 1:**
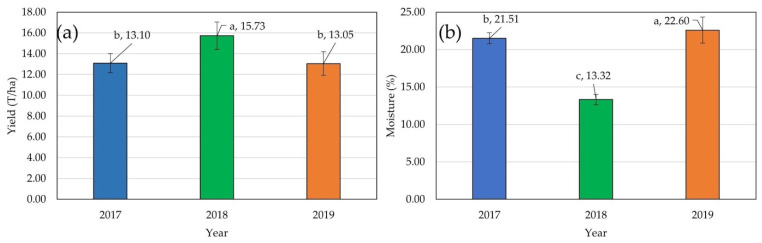
Mean values of agronomic traits: different letters above the columns represent statistically significant differences: (**a**) average scores for grain yield in three seasons; (**b**) average scores for grain moisture in three seasons.

The average grain yield of all evaluated hybrids ranged from 13.05 t ha^−1^ in 2019, 13.10 t ha^−1^ in 2017, to 15.73 t ha^−1^ in 2018. The average grain moisture ranged from 13.32% in 2018, 21.51% in 2017, to 22.60% in 2019 (Figure 1).

A significant effect of the genotype and year was detected for both GY and GM, while the effect of the replication was significant only for GY (Table 1). The effect of block (*p* < 0.005) and genotype by year interaction (*p* < 0.001) were significant only for GM.

**Figure 2:**
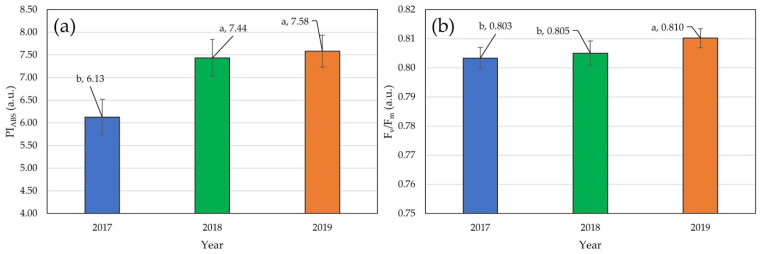
Mean values of fluorescence traits: different letters above the columns represent statistically significant differences: (**a**) average scores for photosynthetic efficiency (PSI_ABS_) in three seasons; (**b**) average scores for quantum yield (F_v_/F_m_) in three seasons.

Significant effects of genotype, year, replication, and block were detected for both F_v_/F_m_ and PI_ABS_ traits (Table 2). The effect of the genotype by year interaction was not significant. The highest mean values for F_v_/F_m_ were recorded in 2019 (0.810) and differed significantly from mean scores in the other two years (0.803 in 2017 and 0.805 in 2018) (Figure 2).

### 2.2. Plateaus of Fluorescence (OJIP)

**Figure 3:**
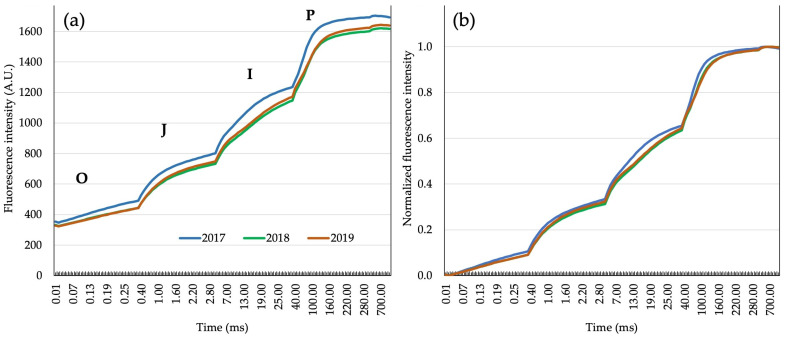
Transients of chlorophyll *a* fluorescence: (**a**) native transients of chlorophyll *a* fluorescence in flowering time across three years and all hybrids; (**b**) normalized transients of chlorophyll *a* fluorescence in flowering time across three years and all hybrids.

Relatively higher fluorescence intensity in all hybrids in 2017 was detected in native data of photosystem II fluorescence induction transients. Maximum fluorescence (F_M_) also showed higher values, unlike zero fluorescence (F_0_). Transient plateaus of the zero fluorescence signal (O), the first plateau of the chlorophyll *a* fluorescence (J), the second plateau of the fluorescence signal (I), and maximum fluorescence (P) were clearly distinguished in the fluorescence induction curve (Figure 3a). Chlorophyll *a* transients were normalized with total variable fluorescence (F_M_-F_0_). As a result, curves from all three years were aligned very close to each other (Figure 3b).

### 2.3. Variance Components Estimated with Mixed Model and Trait Correlations

The variance analysis showed that the components of variance for all factors were larger than zero (Table 3). The variance explained by genotype for GY was higher than the variance explained by the G × E interaction and lower than the error variance. The same scenario was found with PI_ABS_. The variance explained by genotype showed different values for GM: it was lower than the variance explained by G × E interaction (GEI) but higher than the error variance. The same scenario was found with F_v_/F_m_. The heritabilities for all traits were relatively high (0.62 for GM and 0.64 for GY) to high (0.70 for F_v_/F_m_ and 0.84 for PI_ABS_).

The analysis of Pearson’s correlations (Table 4) showed a moderate to weak negative correlation (−0.49) significantly different from zero (*p* < 0.001) between GY and GM. The correlation between GY and PI_ABS_ was positive, weak to very weak (0.16), and significantly different from zero (*p* < 0.05). A weak to very weak negative correlation (−0.20) significantly different from zero (*p* < 0.01) was detected between PI_ABS_ and GM, while the correlation between F_v_/F_m_ and PI_ABS_ was moderate to weak (0.40) and significantly different from zero (*p* < 0.001). A moderate negative genetic correlation (−0.61) was detected between F_v_/F_m_ and GM, while the genetic correlation between F_v_/F_m_ and PI_ABS_ was strong and positive (0.76). All other genetic correlations were weak (−0.10 between GY and GM, 0.35 between GY and F_v_/F_m_, and −0.24 between PI_ABS_ and both GY and GM).

### 2.4. Statistical Models for GY and GM Predictions

**Figure 4:**
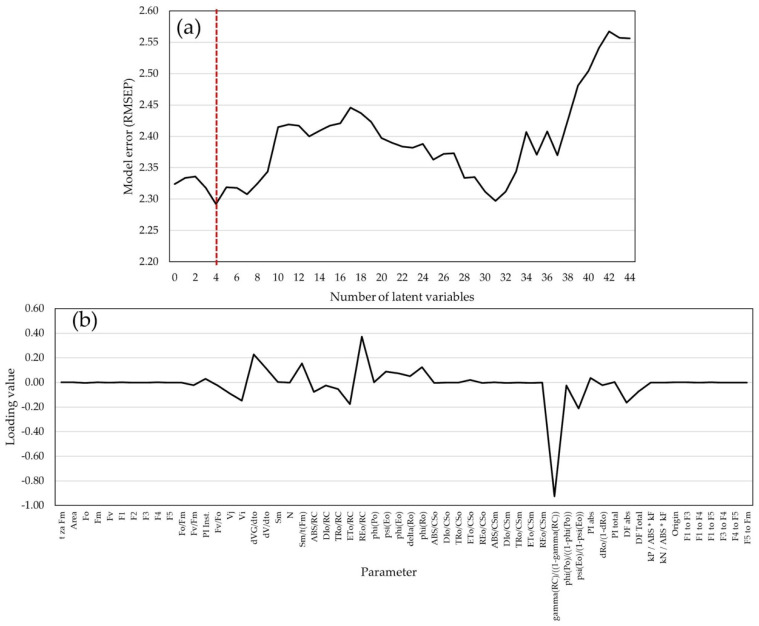
The model for grain yield predictions: (**a**) error of the model (RMSEP) with biophysical parameters of chlorophyll *a* fluorescence as predictors by increasing the number of latent variables (red line shows the lowest RMSEP value); (**b**) coefficients of biophysical parameters of chlorophyll *a* fluorescence in the PLS model for GY prediction with 4 latent variables.

With the increase in the number of latent variables, the increase in the RMSEP (root mean square of predictions) values was determined using 58 biophysical parameters for GY prediction. A total of 44 latent variables (LVs) were estimated, and the lowest RMSEP value (2.292) was scored with 4 LVs (Figure 4a). Therefore, the model with four LVs was used for GY prediction as a calibrated model. The loading values of biophysical parameters in the calibrated model are shown in Figure 4b.

**Figure 5:**
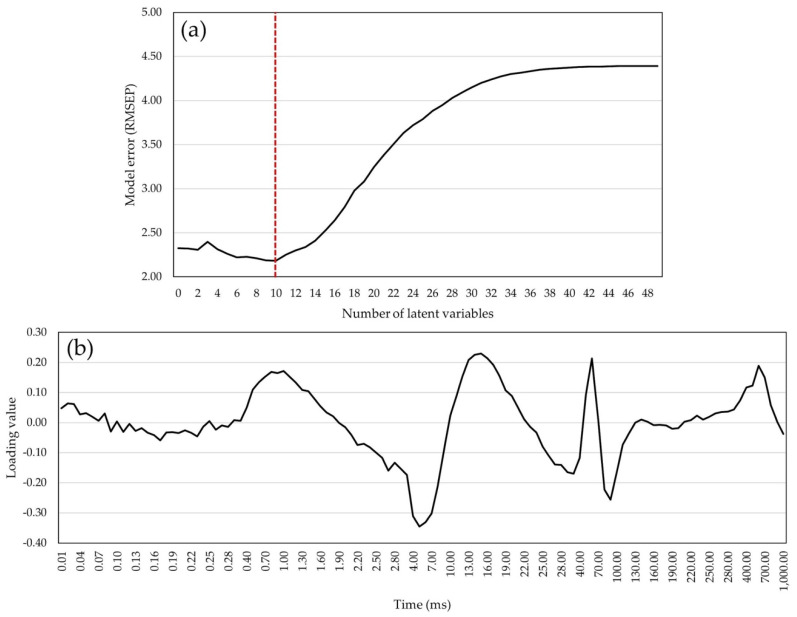
The model for grain yield predictions: (**a**) error of the model (RMSEP) with ChlF transients as predictors by increasing the number of latent variables (red line shows the lowest RMSEP value); (**b**) loading values of ChlF transients in the PLS model with 10 latent variables for GY prediction.

Higher absolute loading values in the model were detected with native chlorophyll *a* fluorescence induction signals (F_m_, F4, and F5), parameters derived from the transient curve (T for Fm, and transient signals from F1 to F5), and cross-section electron flows. In the model with 118 chlorophyll *a* fluorescence transients, RMSEP values decreased until the 10th latent variable and increased until the 48th LV (Figure 5a), after which adding more variables no longer affected the variance explained by the model. The lowest RMSEP value (2.182) was scored in the model with 10 LVs. Pondering values in the model with 10 LVs were higher at J, I, and P steps of the OJIP curve, at the transition of plateau J to plateau I, and during the approximation of values to maximum fluorescence intensity (Figure 5b).

**Figure 6:**
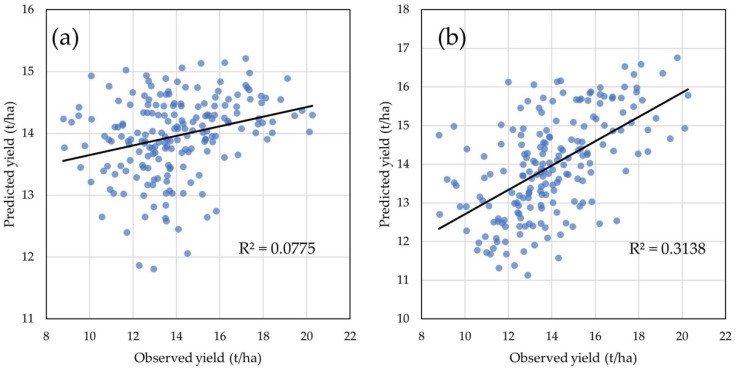
Grain yield predictions: (**a**) GY predicted with the calibrated PLS model using 58 chlorophyll *a* fluorescence biophysical parameters; (**b**) GY predicted with the calibrated PLS model using 118 chlorophyll a fluorescence transients.

The model with four LVs and 58 ChlF biophysical parameters as predictors showed low prediction ability with 7.75% of the total variance for GY explained (Figure 6a). The model with 10 LVs and 118 ChlF transients as predictors explained 31.38% of the total variance for GY (Figure 6b), thus showing higher prediction ability compared to the previous model.

**Figure 7:**
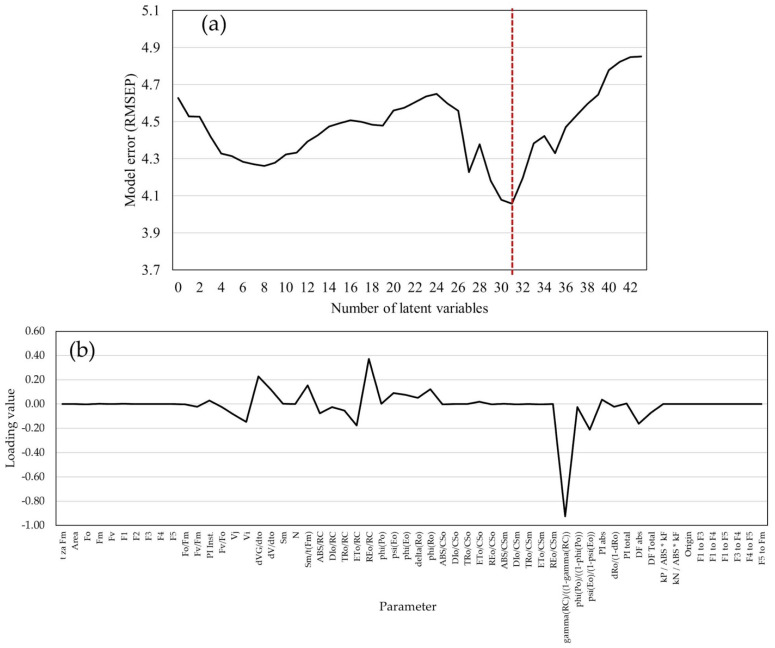
The model for grain moisture predictions: (**a**) error of the model (RMSEP) with biophysical chlorophyll *a* fluorescence parameters as predictors by increasing the number of latent variables (red line shows the lowest RMSEP value); (**b**) loading values of chlorophyll *a* fluorescence biophysical parameters in the PLS model with 4 latent variables for GM predictions.

Using 58 biophysical parameters as predictors for grain moisture during harvest, increasing the number of LVs resulted in unstable RMSEP values. A total of 43 LVs were estimated, and the lowest RMSEP value (4.057) is scored with 31 LVs (Figure 7a); therefore, this model is used for GM predictions at harvest time. Loading values of the GM prediction model with chlorophyll *a* fluorescence biophysical parameters differed from loading values in the GY prediction model (Figure 7b). The highest loading value was scored for $\frac{\gamma_{RC}}{1-\gamma_{RC}}$, which refers to the probability that the chlorophyll molecule of the photosystem II operates as a reaction center. Absolute loading values higher than 0.2 were detected for the parameter $d_{VG}/d_{To}$, which refers to fluorescence curve inclination, and for the parameter $E_{0}/RC$, which refers to the probability that the electron flow of each reaction center reduces the final electron acceptors in photosystem I.

**Figure 8:**
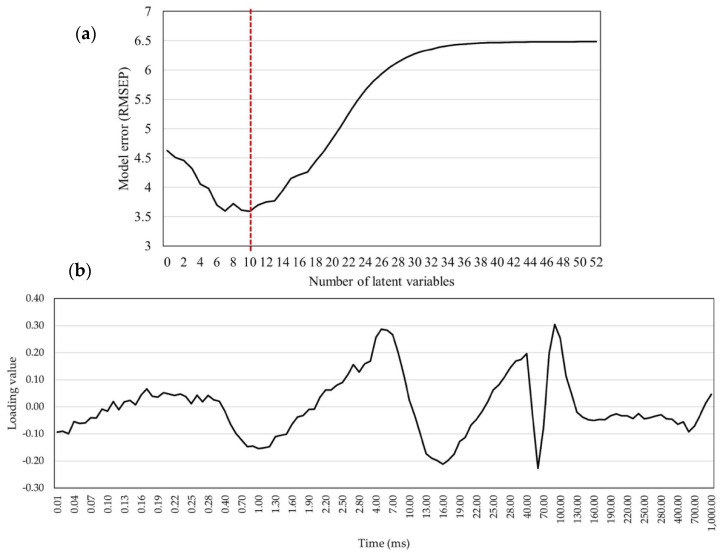
The model for grain moisture predictions: (**a**) error of the model (RMSEP) for GM predictions with chlorophyll a fluorescence transients as predictors by increasing the number of latent variables (red line shows the lowest RMSEP value); (**b**) chlorophyll *a* fluorescence loading values in the PLS model with 10 latent variables for GM predictions.

A total of 52 LVs were estimated in the model with 118 ChlF transients. The RMSEP model values decreased up to the 10th variable and increased from the 10th to the 52nd variable (Figure 8a). The lowest absolute RMSEP value (3.591) was scored in the model with 10 LVs; therefore, this model was used for trait predictions. The pattern of the CaF transient loading values was similar to the pattern in the GY prediction model, with less distinguished values for the P step of the OJIP curve (i.e., maximum fluorescence). The highest absolute loading values in this model were approximately 0.3.

**Figure 9:**
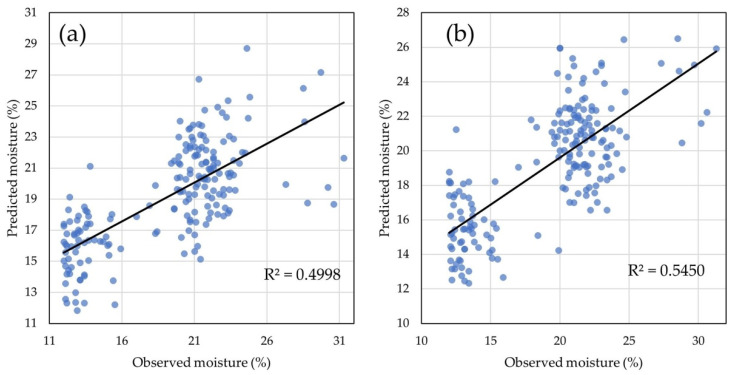
The models for grain moisture predictions: (**a**) GY values predicted with the calibrated PLS model using 58 chlorophyll *a* fluorescence biophysical parameters; (**b**) GM values predicted with the calibrated PLS model using 118 chlorophyll *a* fluorescence transients.

The model with 31 LVs and CaF biophysical parameters as predictors showed moderate prediction ability as it explained 49.98% of the total variance for GM (Figure 9a). The calibrated model with 118 ChlF as predictors of GM showed higher prediction ability as it explained 54.50% of the total variance for GM using only 10 LVs (Figure 9b).

### 2.5. Variance Components of Predicted Traits

Variance component analysis with the mixed model showed components of genetic variance for all predicted traits except the variance of GEI for GY and the genetic variance for GM predicted by using chlorophyll *a* fluorescence transients (Table 5). Considerable fractions of the variance explained by the environment were detected in all predicted traits. The variance explained by GEI was higher than the variance explained by genotype in GM predicted using biophysical parameters and GY predicted using transients. Low to moderate heritability values were scored for all predicted traits except for GM predicted by transients. Genetic correlations with source traits varied from low to very high. In models using biophysical parameters as predictors for GY, moderate to low correlation (0.44) with the source traits was detected. The genetic correlation of GM predicted with biophysical parameters with the source traits was high to very high (0.83) with a very high estimation error (1.05). The highest genetic correlation with the source traits (0.97) was scored in GY predicted by ChlF transients.

**Figure 10:**
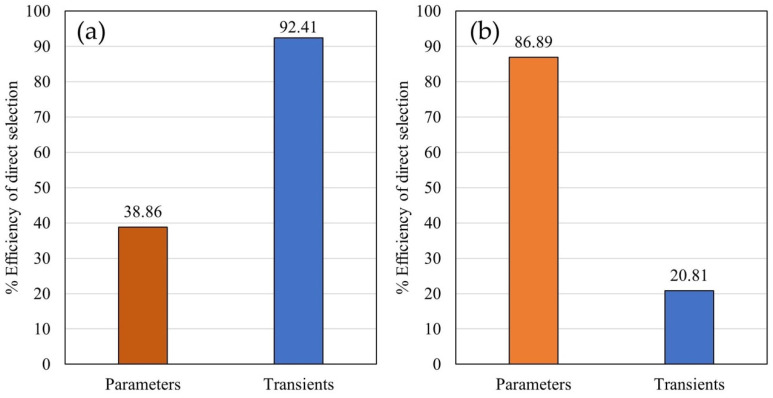
The efficiency of indirect selection: (**a**) efficiency of indirect selection for GY through predicted values using biophysical parameters and ChlF transients; (**b**) efficiency of indirect selection for GM through predicted values using biophysical parameters and ChlF transients.

The efficiency of indirect selection for GY by using values predicted with biophysical parameters was lower (38.86%) compared to efficiency using values predicted with ChlF transients (92.41%) (Figure 10a). The efficiency of indirect selection for GM by using values predicted with biophysical parameters was relatively high (86.89%) and significantly lower by using values predicted with transients (Figure 10b).

## 3. Discussion

### 3.1. Agronomic Traits

The main goal of maize breeding is the development of hybrids with high agronomic performances (high GY, low GM, abiotic and biotic stress tolerance, etc.), which relies on the selection and development of inbred lines from different heterotic pools. GY is calculated on the basis of 14% of GM moisture content. The threshold of 14% of GM is set for practical reasons (e.g., when kernel moisture drops to 14%, the total amount of free water is low; therefore, kernels do not need to dry anymore and are ready to be stored, which significantly lowers production costs). Calculating GY for storage-ready grain ensures that GY is not increased by adding moisture that will eventually evaporate from the kernel. Grain yield increase can be achieved by implementing changes to agrotechnical practices, reducing low and medium-yielding environments, and with progress in breeding that results in more adaptable high-yielding cultivars [52]. It is expected with new hybrids in the future that the difference between low- to medium-yielding environments and high- to very-high-yielding environments will increase while the number of low- to medium-yielding environments is expected to be in a decreasing trend [53]. The average yields at the Agricultural Institute Osijek from 2010 to 2017 were 7.0, 5.7, 4.3, 6.5, 8.1, 6.5, 8.5, and 6.3 t ha^−1^ [54]; therefore, environments on AIO fields can be considered high-yielding environments. The yield increase in China between the 1960s and 2000s [55] was caused by several important factors, with the elite Corn Belt germplasm being probably the most important one. The same examples that showcase the benefits of using elite germplasm can be found around the world; therefore, AIO incorporated the Corn Belt germplasm in its programs decades ago.

This study showed significant differences among years. GY did not differ between 2017 and 2019, while 2018 was significantly different from both other years, which can probably be attributed to above-average temperatures and rapid accumulation of heat units in 2018 that caused shortening of the planting–flowering period for at least seven days compared to 2017 and 2019. Thus, hybrids in the 2018 trials had considerably fewer limitations with growth and development in critical periods (high temperatures and water deficit), which after the kernel maturation reflected a 2.6 t ha^−1^ higher yield than in 2017 and 2019. Favorable temperature patterns that directly influence the accumulation of heat units are one of the key factors in maize production; therefore, high temperatures often lead to vegetation shortening, forced ripening, and yield loss [56]. Significant differences among hybrids across three years were recorded; thus, hybrids in this study can be clustered into two groups: one with higher-yielding hybrids and one with lower-yielding hybrids. Differences between these two groups can be attributed to variations in genotype, year, and replications, which showcases the hybrids with stable yield performance across three years. Significantly lower GM values in 2018 (13.3%) compared to other years (21.5% and 22.6%) can also be explained by the rapid accumulation of heat units in earlier vegetation periods, which sped up maturation and caused lower GM scores at harvest time. The importance of the year for grain moisture was highlighted by the highly significant GEI.

### 3.2. Photosynthetic Efficiency Traits

ChlF analysis offers reliable information on plant status in the field, especially in stressful conditions; therefore, correct interpretation of the ChlF research results through methodology overviews and theoretical fundamentals is essential [57]. ChlF mainly originates from PSII antenna complexes; however, it depends on pigment matrix processes or PSII reaction centers, oxido-reduction reactions on the donor and acceptor side of PSII, and even in the whole electron transfer chain. Based on the photosynthetic apparatus energy flow developed by prof. R. Strasser, known as the JIP-test [58], access to the fluorescence induction curves analysis is explored in detail. One of the main parameters in this study (F_v_/F_m_), the quantum efficiency of primary photochemical processes in PSII, has an important role in maize drought stress research. Along with other parameters (PI, relative leaf water content, and soil water content), Fv/Fm can be used to assess the capacity of the photosynthetic apparatus for collecting solar energy in maize hybrids [59]. Lower values of F_v_/F_m_ and PI indicate that drought stress significantly affects plants in the field, which highlights F_v_/F_m_ as a valuable indicator of drought stress tolerance in maize. Based on such indicators, maize germplasm can be clustered and classified according to drought susceptibility. In this study, the results showcase the importance of fluorescence parameters in grouping hybrids and environments according to quality and stress levels. Fluorescence parameters could be used for predicting GY with statistical models [60], which is one of the main settings of this study (regression model with ChlF traits as predictors and agronomic traits as dependent variables). A number of studies have been carried out to address various environmental effects on ChlF [61,62,63]; therefore, a certain level of predictive ability for maize in field conditions can be attributed to ChlF parameters. In this study, F_v_/F_m_ was used as a predictor of the most important traits in maize selection. Its correlations with GY and GM were very weak positive correlations while genetic correlations with GY and GM were weak positive and moderate negative correlations, respectively. As one of the main ChlF parameters, PI_ABS_ had very weak positive (GY) and negative (GM) correlations with agronomic traits and a very weak genetic correlation with both GY and GM. Determined correlations and variability in both F_v_/F_m_ and PI_ABS_ across three years of research confirmed the findings of many other studies [41,61,62,63], which show an informative assessment of the crop in field conditions and consequently its modeling. High average temperatures in June and July affected the variation in ChlF parameters. This and similar studies linking ChlF parameters with agronomic traits [64] can provide new insights about mechanisms of crop adaptability to high temperatures, thus serving as a reference for the production of abiotic stress-tolerant crops and efficient irrigation management, which is relatively new but an increasingly needed practice considering recent climate changes.

### 3.3. GY Prediction Model Efficiency Analysis

Prediction of grain yield and moisture is one of the main goals of this study. Initially, genetic variability in the ChlF traits in experimental and commercial hybrids enables the identification of genotypes with required photosynthetic traits. Estimating correlations between photosynthetic and agronomic traits and variance structure contributes to a better understanding of the connection between physiological processes and agronomic performances [49]. Furthermore, developing a penalized regression model with ChlF traits as predictors and agronomic traits as dependable variables enables physiologically based GY and GM predictions. These actions enable crossing planning and progeny selection during the season, highlighting resource optimization and shortening the hybrid development process. Finally, indirect selection efficiency estimation via penalized regression model prediction and comparison with direct selection offers guidelines for maize selection and breeding improvement.

Heritability estimates showcase the effect of genetic factors on various traits in a population [65]. Heritability estimates for these traits corresponded with other studies [50,66]. Estimated genetic correlations between agronomic and photosynthetic traits were low; however, due to comprehensive data and sufficient literature sources, only two photosynthetic traits (F_v_/F_m_ and PI_ABS_) were analyzed in terms of variance and genetic correlations, while a full dataset with all parameters and transients was used for model development. RMSEP value increased with an increase in the number of latent variables, which indicates lowering prediction accuracy of the model due to excessive complexity of the model or lack of information for accurate prediction [67]. Selecting the model with four latent variables for GY prediction was aimed at achieving the lowest RMSEP value; however, other factors such as interpretability, simplicity, and practical use, are important in model development. Additional validation of the model was performed in order to confirm its prediction capacity on an independent dataset. The model with 10 LVs that used 118 measured ChlF transients showed higher prediction ability compared to the model with four LVs that used biophysical ChlF parameters. Other studies [46] used ChlF in prediction models to explore the effects of abiotic stresses on plant biomass accumulation in maize. The authors of the study confirmed that using 118 transients showed higher, although comparable, predictive ability than 18 selected JIP parameters. The results of the model efficiency analysis for GY prediction corresponded with other similar studies. In a study about biomass prediction [68], the authors determined that their model explained 7–19% of the total variance, while in a study about agronomic trait predictions through ChlF parameters [49], the authors achieved 35% of the total variance explained for selected agronomic traits. Other studies [69] achieved an accuracy of over 50% in biomass prediction using the combination of normalized difference vegetation indices (NDVIs) and ChlF. Compared to the literature, the results in this study show that the model with 10 LVs has a higher predictive ability than other models, as it explains a higher percentage of the total variance for GY. Furthermore, the results indicate the high value and predictive ability of the model based on measured ChlF transients for agronomic trait predictions in maize.

The importance of different parameters for GY prediction in models is emphasized after pondering analysis. Higher absolute loading values were detected in the model for ChlF induction signals (F_m_, F4, and F5), transient curve parameters (T for F_m_ and transient signals from F1 to F5), and electron flows, which indicates the significance of these factors in explaining GY variability. Furthermore, by adding more LVs, the model analysis with 118 measured ChlF transients showed a gradual decrease in error value (RMSEP). After 48 latent variables, adding more variables had no significant effect on explaining the variance of the model. The lowest RMSEP value (2.182) was scored in the model with 10 LVs, which indicates that this model is reliable for GY predictions. In addition, the loading values in the model with 10 LVs were higher for the J, I, and P steps during the transition from plateau J to plateau I and in convergence with maximum fluorescence intensity. These results indicate the significance of the mentioned parameters in fluorescence efficiency and GY prediction.

### 3.4. GM Prediction Model Efficiency Analysis

Grain moisture during harvest time is the main indicator of technological maturity in maize production. Accurate GM prediction enables optimal harvest planning and, after harvest, maize kernels must not have excessive GM during storage time in order to avoid yield and/or quality losses. In addition, accurate GM monitoring enables optimal decisions regarding harvest time for agronomists and farmers in order to attain the best possible yield and grain quality.

The focus of this study regarding GM traits is the significance of using biophysical ChlF parameters for GM prediction in maize harvest. The model with 31 LVs showed moderate predictive ability, explaining almost 50% of the total variance for GM, which suggests that these parameters can be useful predictors of GM and provide valuable information on technological maturity in maize production. However, the calibrated model with 118 measured ChlF transients showed even better predictive ability. With only 10 LVs, this model explained 54.5% of the total variance for GM at harvest time. This confirms the selection of optimal parameters whose integration into a model can significantly improve GM prediction accuracy. These results can be useful in GM predictions in maize harvest, and provide a solid ground for further research and development of new methods, which could enable accurate monitoring and prediction of the technological maturity of maize, leading to higher GY and quality in maize production.

Research studies on GM prediction in maize harvest are not frequent and are often limited to direct predictions. The limitations of such studies include not considering some important factors such as senescence and plant maturity during development. However, a recent study [70] highlights the significance of using proximal methods in ChlF measurements for predicting the maturity of maize. Proximal methods enable measuring ChlF in plants in real time and on the spot in the field, which provides a more detailed view of the senescence and plant maturity. The results of the study suggest that using proximal methods facilitates the detection of senescence in plants before it is visible with bare eyes. Senescence is the process of biological plant aging and it is associated with maturity; therefore, proximal methods are efficient in maize maturity prediction.

Many studies have addressed the association of water deficit during vegetation and GM in harvest with GY [71,72]. GM prediction at harvest time and moisture dry-down in kernel development [73,74] is an important factor in modern maize breeding and production; nowadays, this factor is even more important due to the transition to earlier maturity hybrids with faster grain moisture dry-down (GMD) in many production regions around the world. Earlier maturity hybrids with shorter vegetation and faster GMD are lowering the costs of seed drying, which is a significant cost in maize production. Proper seed drying management is one of the most significant factors in maize production [72]. Faster GMD can reduce production losses and enable more efficient harvest activities in many different production regions. Further research should be focused on lowering GMD even more in order to exploit the benefits in production and seed drying. In this study, comparable GM prediction values were scored, although the method was applied earlier in the vegetation.

Loading values in the GM prediction model with ChlF biophysical parameters show certain differences compared to loading values in the GY prediction model. The highest loading value was scored for parameter $\frac{\gamma_{RC}}{1-\gamma_{RC}}$, which refers to the probability that chlorophyll molecules in PSII function as a reaction center. Absolute loading values higher than 0.2 were noticed for parameters $\frac{dVG}{dTo}$, which refers to the slope of the fluorescence curve, and $\frac{RE0}{RC}$, which refers to the probability that the electron flow of each reaction center reduces terminal electrone acceptors in PSI. In the model with 118 measured ChlF transients, a total of 52 LVs were estimated. The RMSEP values in the model decreased up to the 10th variable and increased after adding more variables. The loading value pattern of ChlF transients in the GM prediction model was similar to the pattern in the GY prediction model, with somewhat lower values for the P step (maximum fluorescence). The highest absolute loading value in this model was 0.3 approximately. The results of this study showcase the significance of certain ChlF parameters (reaction center probability, fluorescence curve slope, and electron flow reduction probability) in GM predictions at harvest time. Furthermore, the optimal model with LVs exhibited fairly accurate predictions of GM.

### 3.5. Indirect Selection Efficiency Analysis

Indirect selection efficiency (ISE) for GY with biophysical parameters was lower than efficiency with ChlF transients. ISE using biophysical parameters scored 38.86% in the direct selection, while using ChlF transients ISE scored 92.41% efficiency in the direct selection. Therefore, the results suggest that using ChlF transients as predictors would enable a more accurate and more efficient selection of high-yielding genotypes. However, ISE for GM with biophysical parameters was relatively high, scoring 86.89% efficiency of the direct selection, while transients were better in predicting environmental variance, so ISE in this case was 0. These results highlight the significance of the biophysical parameters in predicting GM. We acknowledge a possible perplexity arising from the discrepancy between the grain yield calculation (standardized to 14% moisture) and the selection efficiency results. It is possible that the biophysical parameters are more sensitive to the rate of moisture loss and developmental timing than the final standardized yield. Therefore, even though the final yield is standardized, the parameters that were measured could be more closely related to the process of moisture loss, than the final moisture content.

Many studies have speculated on the usability of the ChlF for indirect selection in maize [41,45,63,75]; however, very few of them analyzed the ISE [49]. The reason for that is the large phenotyping requirements. Phenotyping a large number of plots and collecting reliable information on variance components is a very difficult, if not impossible, task for the majority of the institutions involved in photobiology research. Other secondary traits such as nitrogen use efficiency [14] have been utilized for assessing ISE and improving tolerance to abiotic stress [76]. In a study on drought tolerance and indirect selection in maize [3], costly and time-consuming phenotyping was carried out to explore drought tolerance in maize germplasm. Indirect selection based on the anthesis–silking interval, leaf senescence, leaf chlorophyll content, or GY in controlled conditions did not show higher efficiency than direct selection for GY in drought conditions. Genomic selection for GY under drought with 998 markers scored a relative efficiency of 1.24. Given the possibility of multiple selection marker-based selection cycles per year in maize and the fact that genotyping is more cost-effective than phenotyping for drought tolerance, genomic selection could increase selection gain per unit of time for GY in drought conditions. In this study, with ChlF traits instead of markers, ISE scores were close to the efficiency of the direct selection, which suggests the possibility of faster selection of the paternal inbred lines before flowering, while field crossing is still available. Although indirect selection is relatively less efficient than direct selection, it is used as an additional tool to increase the selection for grain yield [14].

### 3.6. Limitations of This Study

There are several limitations in this study, the first being the modest sample size and limited genetic variability. Furthermore, phenotyping was performed in non-stress conditions, which caused small differences among transients.

## 4. Materials and Methods

### 4.1. Plant Material and Experimental Design

The trial was set at the Agricultural Institute Osijek (AIO) in Osijek, Croatia (45°33′4.00″ N; 18°41′38.00″ E). Osijek is the fourth major city in Croatia, located in eastern Croatia, among four regional centers, Zagreb (capital of Croatia) to the west, Belgrade (capital of Serbia) to the east, Budapest (capital of Hungary) to the north, and Sarajevo (capital of Bosnia and Herzegovina) to the south. This study was conducted across three years (2017–2019) with 16 maize hybrids (belonging to the FAO 400 maturity group, which was recently selected as the most important maturity group in Croatia). Maize hybrids used in this study belong to temperate maize germplasm and have dent-type kernels. Female inbred lines in hybrids H1–H8, H10–H13, and H16 belong to the Iodent heterotic pool, while female inbred lines in hybrid H9 belong to the Iowa Stiff Stalk Synthetic heterotic pool. Male inbred lines were of mixed origin (Ohio 43 and Iowa Stiff Stalk Synthetic) [77,78]. Hybrids H14 and H15 were commercial hybrids of the Agricultural Institute Osijek.

The trial was set as a randomized block design with four replicates. Plot size was 7 m^2^ (two rows, 5 m long with 0.7 m distance between rows), and the total plant density was approximately 71.000 plants per hectare, i.e., 50 plants per plot.

### 4.2. Agrotechnical Practices and Grain Yield

Standard agrotechnical practice for maize was carried out in each season. Wheat was the forecrop in 2017, while in the other two years, the forecrop was barley. After the forecrop harvest, shallow plowing was performed, followed by standard plowing (35 cm) and fertilization (300 kg/ha NPK 7-20-30, plus 100 kg/ha of urea). Each year, all agrotechnical activities were carried out before mid-November. Plowing furrow slice was closed with heavy harrow around February or March, depending on the weather conditions. After sowing preparation, sowing was performed with hand planters, two kernels per hill. Herbicide treatment was applied after sowing and before sprouting with 1 L/ha DualGold 960 EC and 3 L/ha Koban T (Cheminova A/S, Harboør, Denmark). Thinning to one plant per hill was performed in the V6 stage. Inter-row cultivation was performed along with additional fertilization (250 kg/ha KAN). Harvest of the trial plots was performed with a four-row Gleaner F2 combine harvester, which measured plot yield and moisture from the whole sample. GY was calculated with 14% GM as follows:

$$GY(t/ha)=(Plot\;weight\left(kg\right)\times \frac{100-Sample\;moisture(\%)}{86}\times \frac{10,000{\;m}^{2}}{7{\;m}^{2}})/1000$$

Although grain yield and grain moisture are two separate traits, they are also tightly connected, as the GM directly affects the GY (for that reason one of the most important traits in maize breeding and maize production, apart from yield, is grain moisture dry-down). Therefore, GY is calculated by subtracting the GM from the total yield.

The number of days to harvest varied among the years. In 2017, the time span between sowing and harvest was 165 days, in 2018 168 days, and in 2019 158 days. During the vegetation season in 2017, temperatures higher than the long-term average (1961–1990) were measured in June, July, and August [79]. A different temperature pattern was noticed in 2018. Above-average temperatures were measured in April, May, and June, while temperatures at the end of June and the beginning of July were below the long-term average. In 2019, temperatures lower than the long-term average were recorded in May, and temperatures higher than the long-term average were recorded in June, while in July temperatures from both scenarios were recorded. The total amount of precipitation was higher in 2019 compared to the other two seasons. Precipitation accumulation followed a more regular pattern in 2017 and 2019 compared to 2018 with drier conditions in April and May and more rainfall in June and July. The total amount of accumulated rainfall during the seven months of the season was 345 mm in 2017, 460 mm in 2018, and 533 in 2019.

### 4.3. Chlorophyll a Measurements

Chlorophyll *a* fluorescence (ChlF) was measured on three plants per plot during tasseling time in the morning hours (6.30 to 9.00). The central part of the leaf below the ear without a central leaf vessel was used for measurements, after adaptation to dark conditions for 25 to 30 min, using clamps with sealing foam and leaf exposition perforation of 12.56 mm^2^ in diameter with a sliding plate. A hand fluorometer Handy-PEA (Hansatech, UK) was used for measurements. After the dark adaptation period, the leaf surface is illuminated with high-intensity light (650 nm, 3500 μmol m^−2^ s^−1^) in order to induce chlorophyll a fluorescence, which shows a specific increase pattern named O-J-I-P after zero fluorescence stages (O), the first signal plateau of chlorophyll *a* fluorescence (J), the second signal plateau of chlorophyll *a* fluorescence (I), and maximum fluorescence (P) [22,80]. Data of chlorophyll *a* polyphase increase are comprised of 118 directly measured signals (transients), while 56 JIP-test parameters were calculated based on energy flow models. The index of photosynthetic efficiency or performance index (PI_ABS_) of photosystem II is the most sensitive indicator of the plant’s photosynthetic apparatus condition. This index is calculated as follows:

$$PI_{ABS}=\frac{\gamma_{RC}}{1-\gamma_{RC}}\times \frac{\varphi_{Po}}{1-\varphi_{Po}}\times \frac{\psi_{Eo}}{1-\psi_{Eo}}$$

γ_RC_—the probability that the chlorophyll molecule in photosystem II operates as a reaction center.

φ_Po_—maximum quantum yield of the photosystem II (=F_V_/F_M_).

ψ_Eo_—the probability that the excited electron transfers further from plastoquinone $Q_{A}^{-}$

Measurement data were processed with the program PEA Plus ver. 1.10. During the three years of research with 16 maize hybrids in four replications, a total of 32.256 data points of the JIP-test and 67.968 data points of ChlF polyphase increase transients were generated. The full model coefficients and loadings for both transients and biophysical parameters are enclosed in Table S1.

### 4.4. Predictive Statistic Models

Partial least squares (PLS) regression was used for predictive models in this study due to the collinearity between prediction variables. PLS regression is based on data dimensionality reduction by developing synthetic variables with minimal mutual correlation while at the same time aiming to explain the variance in the dependent variable [81].

Synthetic variable development is based on removing shared variance from original variables (penalizing). Each synthetic variable explains as much variance as possible in the original variables (X) and the dependent variable that serves as the model base (y). Unlike the linear regression model y = Xb + e, the PLS algorithm implies that the residual matrix Z derives from data matrices X and y: Z = [X|y].

The PLS program library [67] was used for model development. Two models were developed for the prediction of each trait. The models for GY and GM predictions used 58 biophysical parameters and 118 fluorescence native transients as independent variables (predictors). The models were validated using cross-validation with ten random folds. The number of synthetic variables for calibrated models was determined by Root Mean Square Error of Prediction (RMSEP).

### 4.5. Data Analysis and Model Development

Data analysis in this study was performed in R [82]. The libraries of lme4 [83] and sommer [84] were used for quantitative genetic analysis and variance component estimation. Genetic correlations ($r_{G}$) [76] were estimated as follows:

$$r_{G}=\frac{Cov_{G(XY)}}{\sigma_{G(X)}\sigma_{G(Y)}}$$

where ${Cov}_{G(XY)}$ is the genetic covariance between traits, $\sigma_{G(X)}$ is the genetic variance of the trait X, and $\sigma_{G(Y)}$ is the genetic variance of the trait Y. The efficiency of indirect selection (EI) was estimated as follows:

$$EI=\left|r_{G}\right|h_{X}/h_{Y}$$

where $r_{G}$ is the absolute value of genetic correlation, $h_{X}$ is the square root of the secondary trait heritability, and $h_{Y}$ is the square root of the primary trait of heritability [76].

The heritabilities of traits were estimated on a progeny mean basis from the variance components [65] as follows:

$$H^{2}=\frac{\sigma_{G}^{2}}{\sigma_{G}^{2}+\frac{\sigma_{GE}^{2}}{nE}+\frac{\sigma_{e}^{2}}{nEnR}}$$

where $\sigma_{G}^{2}$ represents the genetic variance component, $\sigma_{GE}^{2}$ represents genotype by environment interaction, $\sigma_{e}^{2}$ is the error variance, and *nE* and *nR* are the number of environments and replications.

The GY and GM for 16 hybrids in three years and four replications (n = 192) were analyzed through multifactorial ANOVA. Fisher’s test of least significant differences was also performed. Standard mean errors were calculated from standard deviation and the square root of the sample number as follows:

$$S.E.=\sigma /\sqrt{n}$$

## 5. Conclusions

The results showed variability in maize germplasm used in this study and highlighted secondary traits as a valuable source of information for in-season predictions of important agronomic traits that can be utilized in maize breeding programs.

## Figures and Tables

**Table 1:** Analysis of variance for GY and GM.

Source of Variation	Df	MS		*p*			
		GY	GM	GY		GM	
Genotype	15	10.2	28.4	<0.001	***	<0.001	***
Year	2	148.1	1618.2	<0.001	***	<0.001	***
Replication	9	10.5	1.3	<0.001	***	0.132	n.s.
Block	9	4.4	2.2	0.094	n.s.	0.005	**
Genotype:Year	30	3.9	9.2	0.065	n.s.	<0.001	***
Error	126	2.6	0.8				

Symbols **, and *** represent significance with *p* = 0.01, and *p* = 0.001. Tag n.s. (not significant) means that there is no significant effect to the trait.

**Table 2:** Analysis of variance for F_v_/F_m_ and PI_ABS_.

Source of Variation	Df	MS		*p*			
		F_v_/F_m_	PI_ABS_	F_v_/F_m_		PI_ABS_	
Genotype	15	0.00010	1.8	<0.001	***	<0.001	***
Year	2	0.00083	41.3	<0.001	***	<0.001	***
Replication	9	0.00014	1.1	<0.001	***	<0.001	***
Block	9	0.00006	0.6	0.046	*	0.026	*
Genotype:Year	30	0.00003	0.3	0.383	n.s.	0.493	n.s.
Error	126	0.00003	0.3				

Symbols * and *** represent significance with *p* = 0.05 and *p* = 0.001. Tag n.s. (not significant) means that there is no significant effect to the trait.

**Table 3:** Components of variance for GY, GM, Fv/Fm, and PI_ABS_.

Components of Variance	GY	GM	F_v_/F_m_	PI_ABS_
σ^2^_G_	0.553	1.296	5.88 × 10^−6^	0.120
σ^2^_E_	2.124	25.335	1.02 × 10^−5^	0.622
σ^2^_GxE_	0.307	2.209	7.17 × 10^−5^	0.003
σ^2^_e_	2.593	0.795	2.97 × 10^−5^	0.272
*H* ^2^	0.64 ± 0.17	0.62 ± 0.17	0.70 ± 0.13	0.84 ± 0.07

σ^2^_G_ is the variance explained by genotype, σ^2^_E_ is the variance explained by environment, σ^2^_GxE_ is the variance explained by the genotype by environment interaction, σ^2^_e_ is the model error variance, and *H*^2^ is heritability.

**Table 4:** Coefficients of Pearson’s correlations (above the diagonal) and genetic correlations (below the diagonal) calculated from the genetic variance–covariance matrix and standard error.

r	GY	GM	F_v_/F_m_	PI_ABS_
GY	–	−0.49 ***	0.06	0.16 *
GM	−0.10 ± 0.42	–	0.08	−0.20 **
F_v_/F_m_	0.35 ± 0.37	−0.61 ± 0.30	–	0.40 ***
PI_ABS_	−0.24 ± 0.35	−0.24 ± 0.19	0.76 ± 0.20	–

Symbols *, **, and *** represent significance with *p* = 0.05, *p* = 0.01, and *p* = 0.001.

**Table 5:** Variance components, heritabilities, and correlations with source traits for predicted values of GY and GM.

Variance Component	Yield–Parameters	Moisture–Parameters	Yield–Transients	Moisture–Transients
σ^2^_G_	0.036	0.29686	0.080	0.032
σ^2^_E_	0.191	8.053031	1.156	10.087
σ^2^_GxE_	0.001	0.81675	0.137	1.650
σ^2^_e_	0.233	4.19772	0.409	2.996
*H* ^2^	0.65 ± 0.16	0.32 ± 0.28	0.50 ± 0.23	0.03 ± 0.44
r_G_	0.44 ± 0.45	0.83 ± 1.05	0.97 ± 0.36	0.11 ± 0.63

## Data Availability

Data is available from corresponding authors upon request.

## Supplementary Materials

The following supporting information can be downloaded at: https://www.mdpi.com/article/10.3390/plants14081216/s1, Table S1: The full model coefficients and loadings for both transients and biophysical parameters.
