# Supplementary material for: In-Season Predictions Using Chlorophyll a Fluorescence for Selecting Agronomic Traits in Maize
Source: Plants (Basel). 2025 Apr 15;14(8):1216. doi: 10.3390/plants14081216 (PMC12030001; doi:10.3390/plants14081216)
Supplement: Supplementary file 1 [file plants-14-01216-s001.zip › plants-3470705-supplementary.pdf]

Supplementary file

Table S1: The full model coefficients and loadings for both transients and biophysical parameters.

Transients	Moisture	Grain yield
F1	0,004437862	-0,02680635
F2	0,056808918	-0,077652094
F3	0,011009425	-0,049680714
F4	-0,001770531	-0,033314401
F5	0,026881607	-0,042384511
F6	0,007107873	-0,089763332
F7	-0,000161926	-0,017326552
F8	0,048902	-0,033621131
F9	-0,030285168	-0,007137631
F10	0,010563704	0,010095621
F11	0,005859631	0,033744714
F12	-0,003824352	-0,021961958
F13	-0,018757879	0,011812489
F14	0,042520789	0,000867672
F15	-0,007200408	-0,018914467
F16	0,00054723	0,026427486
F17	-0,040024094	0,074868231
F18	-0,017765201	0,02200436
F19	-0,001329726	0,002313694
F20	-0,00727892	0,067652721
F21	-0,002975891	0,031081445
F22	-0,005119405	0,001599477
F23	-0,026755766	0,010782996
F24	-0,002690968	0,000855807
F25	0,003178505	-0,017001708
F26	0,010455008	-0,011850394
F27	0,010487603	-0,031343294
F28	0,002046867	0,020656421
F29	-0,007368004	0,013470431
F30	0,013269984	-0,017784296
F31	-0,005205657	-0,000683542
F32	0,001414914	0,00591573
F33	0,005121417	-0,015645807
F34	0,010177398	-0,071481152

F35	0,005528015	-0,019466079
F36	0,021140323	-0,013526289
F37	0,005664259	-0,014115197
F38	0,018025011	-0,035303299
F39	0,00201969	-0,044074246
F40	0,00076826	-0,015197991
F41	-0,008750229	-0,009729635
F42	-0,006431334	-0,033636375
F43	-0,012712994	-0,010410501
F44	0,01677151	-0,025956594
F45	0,011876108	-0,0175347
F46	0,018473787	-0,024387925
F47	-0,000943024	-0,057011453
F48	-0,003443512	0,013140111
F49	-0,007698453	0,03036365
F50	-0,006120231	0,004752219
F51	0,00526555	0,017103752
F52	0,007551941	0,006070762
F53	0,012055142	0,020336204
F54	-0,03287291	0,121206259
F55	-0,012502823	0,01842333
F56	-0,013074323	0,078803235
F57	-0,041973858	0,075589418
F58	-0,093528115	0,138832281
F59	-0,06087781	0,107326384
F60	-0,011667074	0,006852981
F61	0,017197881	-0,056962376
F62	0,03417794	-0,069210724
F63	0,038559127	-0,063254884
F64	0,041804873	-0,053751353
F65	0,030893671	0,006752602
F66	0,015973057	0,007074145
F67	-0,006026693	0,008053548
F68	0,027354281	-0,004866744
F69	0,031502427	0,048653144
F70	-0,022787092	0,019260634
F71	-0,003966302	0,012174651
F72	-0,031273707	0,014654589
F73	-0,027567468	0,010729085
F74	0,004347135	-0,004139497
F75	-0,009578505	0,002727302

F76	-0,02790819	0,008780966
F77	-0,012777216	-0,027495765
F78	0,012032496	0,009373895
F79	0,001213164	-0,006319863
F80	-0,015632769	-0,014872876
F81	-0,012376107	-0,037142924
F82	-0,000422507	0,037595154
F83	-0,001618449	-0,003991624
F84	0,009220341	-0,002884649
F85	0,029505205	0,020475774
F86	0,027275723	-0,025348085
F87	0,041295255	-0,078822857
F88	-0,056531457	0,028107974
F89	-0,053435429	0,116303382
F90	-0,010541296	0,082071173
F91	0,025854764	0,035668966
F92	0,043788384	-0,125151951
F93	0,043454167	-0,135481327
F94	0,034535105	-0,124021004
F95	0,03355954	-0,071852516
F96	0,018312529	-0,045598053
F97	-0,002075893	-0,043741795
F98	-0,005651113	-0,001429544
F99	-0,005444556	0,009288877
F100	-0,031165368	0,016908969
F101	-0,019284799	0,049675017
F102	-0,015040883	0,033251156
F103	-0,004860832	0,042863999
F104	-0,017245932	0,054061113
F105	-0,022173578	0,07087676
F106	-0,037367875	0,017015869
F107	-0,004881284	0,035522867
F108	-0,007642164	0,035025941
F109	-0,019090437	0,065861766
F110	-0,02267477	0,032647087
F111	0,003976228	0,068113377
F112	0,03044477	-0,031065061
F113	0,018832784	0,013702359
F114	0,009399313	-0,011891669
F115	0,013932436	-0,013779391
F116	0,006820433	-0,05843192

F117	0,004467204	-0,030705453
F118	0,002912039	-0,069754714

Transients	Loading weight (Grain yield)	Loading weight (Moisture)
F1	-0,012641924	-0,035730991
F2	0,140082217	-0,118084432
F3	0,009678212	-0,084263057
F4	-0,050354982	-0,032253761
F5	0,050701661	-0,057048861
F6	-0,015626362	-0,139593587
F7	-0,025681985	-0,02025523
F8	0,113666451	-0,029937761
F9	-0,08877171	-0,034434195
F10	0,008386247	0,031069507
F11	-0,01610188	0,08416725
F12	-0,044341015	-0,019755633
F13	-0,085441595	0,034974066
F14	0,112091551	0,019644052
F15	-0,029173807	-0,028940633
F16	-0,012291491	0,058028787
F17	-0,154596192	0,159816699
F18	-0,076912345	0,058691638
F19	0,001485714	-0,002012623
F20	-0,026327863	0,131091543
F21	0,002913707	0,039341841
F22	-0,001823534	-0,005434809
F23	-0,082438482	0,025559488
F24	-0,009630957	0,012829754
F25	0,01941226	-0,030612887
F26	0,046048503	-0,024139409
F27	0,058409439	-0,066355346
F28	0,025460817	0,03478772
F29	-0,007593925	0,025991976
F30	0,055686414	-0,025069214
F31	0,027460538	-0,009408123
F32	0,082207169	-0,027505388
F33	0,098133134	-0,067896712
F34	0,082853885	-0,133235917
F35	0,113247315	-0,079077569
F36	0,145560883	-0,049070237

F37	0,08826712	-0,04568147
F38	0,121863875	-0,081405043
F39	0,070088293	-0,099942911
F40	0,057348316	-0,046088283
F41	-0,001279078	-0,017012335
F42	0,008177301	-0,063809183
F43	-0,020156317	-0,021206976
F44	0,043034473	-0,024302879
F45	0,051117408	-0,043050022
F46	0,041082862	-0,030076136
F47	-0,024128525	-0,091341144
F48	-0,021257267	0,01715866
F49	-0,041052564	0,051728147
F50	-0,034973422	-0,010547581
F51	0,00117872	0,004660241
F52	-0,008727437	0,004642062
F53	0,020117755	0,008749937
F54	-0,110701915	0,175050518
F55	-0,074516237	0,012399907
F56	-0,057085024	0,09689334
F57	-0,167874747	0,112600716
F58	-0,321367827	0,223485005
F59	-0,290526741	0,275369825
F60	-0,208789852	0,196774374
F61	-0,169921414	0,148814604
F62	-0,127758569	0,1332552
F63	-0,062958387	0,080722739
F64	0,004669084	0,019731485
F65	0,034017177	0,057424527
F66	0,057647159	-0,020055356
F67	0,032805754	-0,064736642
F68	0,185989759	-0,139615958
F69	0,228640401	-0,06312741
F70	0,062222326	-0,118433332
F71	0,133241908	-0,134228572
F72	0,040904656	-0,122505732
F73	0,044852666	-0,118081085
F74	0,135719148	-0,128964609
F75	0,060850391	-0,086116524
F76	-0,011738488	-0,053937883
F77	0,006522958	-0,089481875

F78	0,05774657	0,012846258
F79	0,015420508	-0,012763134
F80	-0,057703352	-0,008327835
F81	-0,076956406	-0,013789774
F82	-0,061372919	0,142188237
F83	-0,089586507	0,090913788
F84	-0,084870571	0,123328518
F85	-0,153586892	0,280476542
F86	-0,12480464	0,144003801
F87	0,156104358	-0,187687504
F88	0,034006638	-0,169030804
F89	0,001532555	0,039750901
F90	-0,046961487	0,173048133
F91	-0,11111986	0,282834256
F92	-0,127837929	0,072566513
F93	-0,127951882	0,046377624
F94	-0,110006023	0,016863209
F95	-0,065368112	0,053176735
F96	-0,075628297	0,05569724
F97	-0,089368493	-0,000177605
F98	-0,050470944	0,016063059
F99	-0,030841206	0,01563326
F100	-0,091821685	0,002210639
F101	-0,021543718	0,024575852
F102	-0,004585073	-0,012604568
F103	0,047899801	-0,015960709
F104	0,014299056	-0,000956911
F105	0,017198309	0,009894384
F106	-0,026699096	-0,092602643
F107	0,084437474	-0,066951882
F108	0,066470512	-0,058463792
F109	0,043020935	-0,018234583
F110	0,046276866	-0,091709868
F111	0,126035793	-0,021643395
F112	0,220827229	-0,206054948
F113	0,153866679	-0,069691567
F114	0,096142651	-0,064528908
F115	0,082487597	-0,016652684
F116	0,053604178	-0,060778305
F117	0,027331864	0,029007575
F118	-0,006955384	0,003894218

Parameters	Grain yield	Grain moisture
t.for.Fm	-0,024320414	-0,048644834
Area	0,000325881	0,618556464
Fo	-0,000230263	35,11909008
Fm	0,002124111	-77,78953959
Fv	0,002354421	56,60239766
F1	-1,42E-05	0,317664896
F2	0,000133172	-0,26983398
F3	0,000161326	3,918933159
F4	0,001691157	30,76976259
F5	-0,000748082	10,4973155
Fo.Fm	-4,57E-07	-9,43739392
Fv.Fm	4,62E-07	-1,729695932
PI.Inst.	-4,71E-06	-53,1410432
Fv.Fo	1,18E-05	30,78947897
Vj	1,17E-06	185,6367568
Vi	-1,35E-06	125,2054741
dVG.dto	6,67E-07	37,46480663
dV.dto	2,62E-07	-249,1533879
Sm	-0,000100397	-1,806783683
N	-0,000290053	-0,189118154
Sm.t.Fm.	1,87E-06	-249,6644741
ABS.RC	-5,08E-06	-512,2486469
DIo.RC	-1,63E-06	-175,7483078
TRo.RC	-3,46E-06	-355,9883032
ETo.RC	-3,72E-06	-94,12265277
REo.RC	7,92E-07	1873,332417
phi.Po.	4,57E-07	9,43739392
psi.Eo.	-1,17E-06	-185,6367568
phi.Eo.	-6,00E-07	-175,3466483
delta.Ro.	2,91E-06	-277,1566843
phi.Ro.	1,30E-06	-82,1564131
ABS.CSo	-0,000230263	35,11909008
DIo.CSo	-0,000173363	18,74518947
TRo.CSo	-5,69E-05	1,02899646
ETo.CSo	-0,000311976	1,889388827
REo.CSo	0,000306845	-13,29531562
ABS.CSm	0,002124111	-77,78953959
DIo.CSm	-0,000230263	35,11909008

TRo.CSm	0,002354421	56,60239766
ETo.CSm	0,000432977	32,05749751
REo.CSm	0,0028722	12,4341756
gamma.RC....1.gamma.RC..	2,55E-06	485,0322045
phi.Po....1.phi.Po..	1,18E-05	30,78947897
psi.Eo...1.psi.Eo..	-2,04E-05	89,61911638
PI.abs	-5,63E-06	16,12796082
dRo..1.dRo.	1,39E-05	18,70380931
PI.total	7,66E-05	-1,074372111
DF.abs	-9,05E-09	-108,1463349
DF.Total	5,18E-06	-294,4172247
kP...ABS...kF	1,05E-08	16,84671156
kN...ABS...kF	2,62E-09	9,415465163
Origin.to.F1	9,31E-05	0,70476605
F1.to.F3	0,000438724	-2,233598947
F1.to.F4	0,002437288	1,213576324
F1.to.F5	0,000250262	-1,507867697
F3.to.F4	0,001949943	-0,109463186
F4.to.F5	-0,002128175	0,885566063
F5.to.Fm	-2,60E-05	-0,61629685

**Parameters**	**Grain yield**	**Grain moisture**
TRo.CSm	-0,11660698	0,041268768
ETo.CSm	-0,068116721	0,036594994
REo.CSm	0,153436508	0,027727066
gamma.RC....1.gamma.RC..	0,000144358	-0,757545999
phi.Po....1.phi.Po..	0,00022032	-0,031759886
psi.Eo...1.psi.Eo..	-0,000252431	-0,179115325
PI.abs	0,001302083	0,066498803
dRo..1.dRo.	0,000908058	-0,024024286
PI.total	0,006919204	0,005010234
DF.abs	0,000102311	-0,123528835
DF.Total	0,000460637	0,02585881
kP...ABS...kF	6,24E-07	0,008588404
kN...ABS...kF	1,76E-07	0,002370042
Origin.to.F1	-0,004332231	0,000203422
F1.to.F3	-0,026173878	0,000117904
F1.to.F4	-0,12138053	-0,000230172
F1.to.F5	-0,011675596	0,001070974
F3.to.F4	-0,097020416	-0,000676684

F4.to.F5	0,111821157	-0,000378439
F5.to.Fm	0,008667266	0,004443712
